# Transient Reduction of FMD-Response and L-Arginine Accompanied by Increased Levels of E-Selectin, VCAM, and ICAM after Prolonged Strenuous Exercise

**DOI:** 10.3390/sports9060086

**Published:** 2021-06-17

**Authors:** Christoffer Nyborg, Helene Støle Melsom, Martin Bonnevie-Svendsen, Jørgen Melau, Ingebjørg Seljeflot, Jonny Hisdal

**Affiliations:** 1 Institute of Clinical Medicine, Faculty of Medicine, University of Oslo, 0318 Oslo, Norway; h.s.melsom@studmed.uio.no (H.S.M.); martin.bonnevie@gmail.com (M.B.-S.); jorgen@melau.no (J.M.); uxinlj@ous-hf.no (I.S.); jonny.hisdal@medisin.uio.no (J.H.); 2 Department of Vascular Surgery, Oslo University Hospital, 0424 Oslo, Norway; 3 Department of Prehospital Care, Vestfold Hospital Trust, 3103 Toensberg, Norway; 4 Center for Clinical Heart Research, Department of Cardiology, Oslo University Hospital, 0424 Oslo, Norway

**Keywords:** FMD, endothelium, NO, inflammation, ironman, triathlon, E-selectin, VCAM-1, ICAM-1, L-arginine

## Abstract

We assessed endothelial function by flow-mediated dilatation (FMD), levels of the NO-precursor L-arginine, and markers of endothelial inflammation before, at the finish line, and one week after the Norseman Xtreme triathlon. The race is an Ironman distance triathlon with a total elevation of 5200 m. Nine male participants were included. They completed the race in 14.5 (13.4–15.3) h. FMD was significantly reduced to 3.1 (2.1–5.0)% dilatation compared to 8.7 (8.2–9.3)% dilatation before the race (*p* < 0.05) and was normalized one week after the race. L-arginine showed significantly reduced levels at the finish line (*p* < 0.05) but was normalized one week after the race. Markers of endothelial inflammation E-Selectin, VCAM-1, and ICAM-1 all showed a pattern with increased values at the finish line compared to before the race (all *p* < 0.05), with normalization one week after the race. In conclusion, we found acutely reduced FMD with reduced L-arginine levels and increased E-Selectin, VCAM-1, and ICAM-1 immediately after the Norseman Xtreme triathlon. Our findings indicate a transient reduced endothelial function, measured by the FMD-response, after prolonged strenuous exercise that could be explained by reduced NO-precursor L-arginine levels and increased endothelial inflammation.

## 1. Introduction

Flow-mediated dilatation (FMD) refers to the non-invasive assessment of endothelial function by studying the brachial artery’s dilatation in response to increased blood flow [1]. The dilatation is endothelial-dependent [2] as increased shear forces from the blood activate mechanoreceptors in the endothelium [3], causing a predominantly NO-mediated relaxation of the arterial smooth muscles [4].

There are conflicting reports on the acute changes of FMD after exercise [5,6,7,8,9,10,11,12,13,14,15,16]. Still, a biphasic response has been proposed with an initial reduction in FMD after exercise with normalization within 24–48 h [17]. The initial FMD after exercise has also been shown to be inversely correlated to the exercise intensity [5]. However, there are few studies of how prolonged exercise affects the FMD-response [6,16,17] and as far as we are aware there are no studies of FMD after any Ironman distance triathlon.

Prolonged strenuous exercise, as in Ironman distance triathlons, is known to cause a multitude of physiological changes that may influence the endothelial function, including a state of inflammation [18,19,20], increased cardiac- and muscular-damage biomarkers [18,19,20,21,22], and altered bioavailability of essential nutrients [23]. The main aim of the present study was to elucidate the acute effect of such extreme strenuous physical activity on vascular function.

We hypothesized that prolonged strenuous exercise affects the endothelium’s inflammatory state and L-arginine’s bioavailability, a precursor of the NO-signaling molecule [24]. To assess the endothelial inflammation, we measured E-selectin, ICAM-1, and VCAM-1, which are adhesion molecules expressed on the endothelial cells, stimulated by circulating inflammatory cytokines [25] and associated with endothelial damage [26].

## 2. Materials and Methods

### 2.1. Study Population

The study was conducted on participants in Norseman Extreme Triathlon 2019. The competition started with a jump from a ferry and a 3800 m swim in the Hardanger Fjord, followed by 180 km bicycling, with an elevation of more than 3000 m before finishing off with a 42.2 km run to Mt. Gaustatoppen (1883 m above sea level). The total elevation of the racecourse was 5200 m.

The study was announced through an email to participants, and interested participants were sent more information about the study before they decided to enroll. The day before the race start, willing male participants who could perform a follow-up measurement at Oslo University Hospital one week after the race were included in the study. Male participants were selected because we could not control for menstrual cycles due to fixed race dates [27]. The participants were examined the day before the race, as soon as possible after crossing the finish line, and one week after the race.

The Regional Committee for Medical and Health Research Ethics in Norway (REC) approved all experimental measurements (reference: 2016/932), and the study was conducted according to the Declaration of Helsinki. All participants gave written informed consent.

### 2.2. Flow-Mediated Dilatation (FMD)

Participants were examined in the supine position after 20 min of rest. A standard blood pressure cuff was positioned around the right arm, two inches below the antecubital fossa, and the brachial artery was imaged 5–9 cm above the ante-cubital fossa. A linear array transducer probe (9 MHz, GE Vivid E95, GE Healthcare, Chicago, IL, USA) was used to acquire the brachial artery’s images. The transducer was placed in a fixed position at the brachial artery and stabilized with a custom-made tripod. The cuff was inflated to a supra-systolic pressure (230 mmHg) for 5 min. B-mode images and pulsed doppler measurements of the right brachial artery were continuously captured for 190 s, starting 10 s before the cuff was deflated. Automated analysis of end-diastolic diameters and blood velocities was performed in an edge-detecting software (Brachial analyzer, Medical Imaging Applications LLC, Coralville, IA, USA). FMD was calculated as the percentage increase in diameter from 10 s before cuff deflation to maximum dilatation post-occlusion. The maximum flow was calculated from blood velocities and diameter measurement as a measurement of stimuli.

### 2.3. Blood Samples

Venous blood samples were collected at all time points in vacuum containers with silica particles and gel separators. The blood was clotted for 30 min at room temperature, serum was separated by centrifugation at 2000× *g* for 10 min within 1 h, and the serum was pipetted to separate freeze-tolerant containers. All samples were transported on ice to freezing storage with a temperature of −80 °C. Commercial ELISA kits were used for VCAM-1, ICAM-1, and E-selectin (R&D Systems Europe, Abingdon, Oxon, UK). Intra assay coefficients of variations (CVs) were 2.1%, 2.8%, and 2.2%, respectively. L-arginine was determined by high-performance liquid chromatography (HPLC) and pre-column derivatization with o-phthaldialdehyde (OPA) (Sigma Chemicals Co, St Louis, MO, USA).

### 2.4. Statistics

Friedman’s tests were applied to examine for any differences between the times of measurements for each test. Wilcoxon Signed-Rank Sum tests were applied as post hoc tests. A *p*-value of <0.05 was considered significant. All values are presented as median (1. quartile, 3. quartile). Statics was conducted in R (R version 4.0.3, R Foundation for Statistical Computing, Vienna, Austria).

## 3. Results

### 3.1. Subjects

Nine male participants of the Norseman Xtreme Triathlon were included in this study. Complete sets of blood samples were collected in eight subjects. The subjects did not compete in any long-distance running or triathlon competition the week between the measurements. Characteristics of the subjects are given in Table 1.

### 3.2. Flow-Mediated Dilation (FMD)

**Figure 1:**
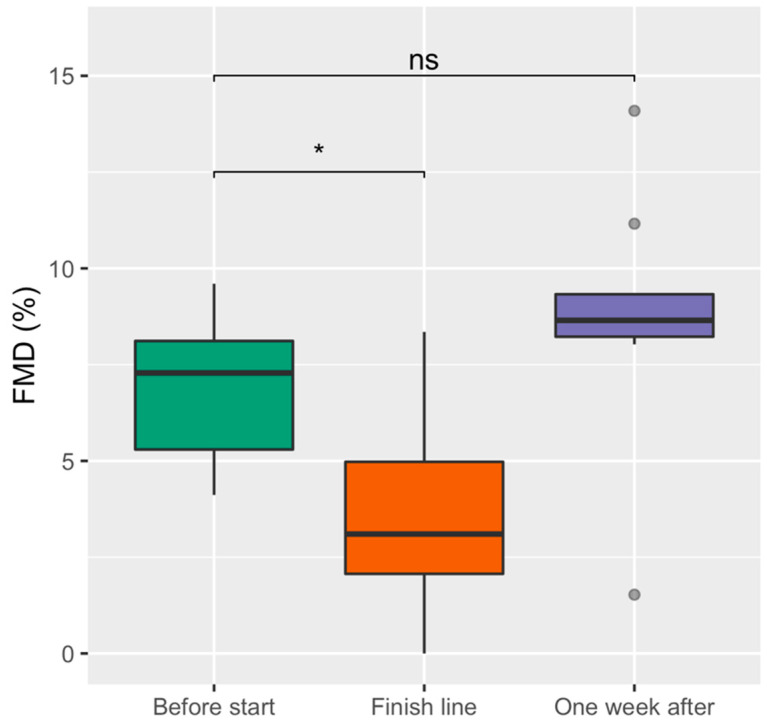
Flow-mediated dilatation (FMD) before the start, at the finish line, and one week after the race with two-sided post hoc Wilcoxon Signed-Rank Sum tests. The boxplots are drawn with a thick line as median; boxes are the interquartile range (IQR) from 1. quartile to 3. quartile; whiskers are maximum value within 1.5 times IQR; values above or below 1.5 times IQR are shown as outliers. * Indicates *p*-value < 0.05 and ns a *p*-value > 0.05. Friedman’s Ranked Sum test showed a *p*-value < 0.05.

As seen in Figure 1, FMD was reduced at the finish line compared to baseline: 3.1 (2.1–5.0) vs. 8.7 (8.2–9.3), *p* < 0.05). The FMD returned to baseline values one week after the competition (*p* = 0.25, compared to before start). There were no significant differences between arterial baseline diameters (*p* = 0.24) or maximum flow calculations (*p* = 0.10) between the time points.

### 3.3. Biomarkers

**Figure 2:**
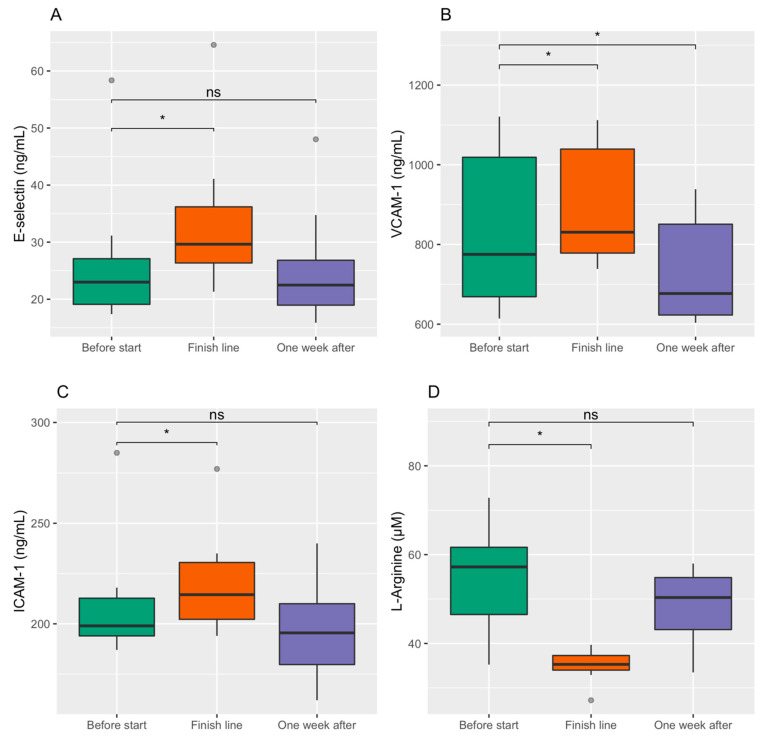
E-selectin (**A**), VCAM-1 (**B**), ICAM-1 (**C**), and L-Arginine (**D**) before the start, at the finish line, and one week after the race with two-sided post hoc Wilcoxon Signed-Rank Sum tests. The boxplots are drawn with a thick line as median; boxes are the interquartile range (IQR) from 1. quartile to 3. quartile; whiskers are maximum value within 1.5 times IQR; values above or below 1.5 times IQR are shown as outliers. * Indicates *p*-value < 0.05 and ns a *p*-value > 0.05. Friedman’s Ranked Sum test for all the biomarkers showed a *p*-value < 0.05.

E-Selectin, VCAM-1, and ICAM-1 all showed a similar pattern with increased values at the finish line compared to before the race (all *p* < 0.05) and reduction one week after the race. E-selectin returned to baseline values one week after the race (*p* = 0.74). VCAM-1 was reduced below baseline values one week after (*p* < 0.05). ICAM-1 showed a similar trend to VCAM-1, but levels after one week were not statistically significantly different from the baseline (*p* = 0.06). L-arginine showed significantly reduced levels at the finish line (*p* < 0.05) and was normalized one week after the race (*p* = 0.08). Full results for E-Selectin, VCAM-1, ICAM-1, and L-arginine are shown in Figure 2. Raw data are also made available in the Supplemental information as S1.

## 4. Discussion

This study’s main finding was that triathletes participating in the Norseman Xtreme triathlon showed a transient reduction in FMD-response immediately after the race. The FMD-response was normalized within one week after the competition. Secondly, we found corresponding reduced levels of the NO-precursor L-arginine after the race [24]. In addition, we observed increased levels of the endothelial inflammation markers, E-Selectin, VCAM-1, and ICAM-1, after the race [25]. The alterations in L-arginine, E-selectin VCAM-1, and ICAM-1 were all transient, like the changes in FMD.

Reports of changes in FMD after prolonged exercise are scarce. One study by Dawson et al. after the London Marathon in 2007 demonstrated a reduced femoral FMD but no significant changes in brachial FMD [6]. However, Dawson and Green, Cable, and Thijssen later proposed in a synthesis on effects of acute exercise on FMD in 2013 that FMD is acutely decreased immediately after exercise, followed by an improvement 1–24 h postexercise and normalization by 24–48 h [17]. Following this thesis, the measurements’ timing could explain the lack of observed changes in systemic endothelial function measured by brachial FMD after the London Marathon. Simultaneously, reduced FMD in the femoral artery among the marathon runners indicates more long-lasting changes in the endothelial function in the conduit arteries supplying the lower body’s active muscle bed [6]. In triathlons, the upper body muscles are used in swimming and partly statically during biking, causing active muscle usage in both legs and arms during the exercise event. The legs are most affected [28], but the use of the upper body causes the brachial artery to be subjectable to both local and systemic effects regarding increased blood flow and shear, in contrast to after a marathon where the brachial artery is mostly subjectable to systemic effects only.

Another potential explanation for the brachial FMD reduction after the Norseman Xtreme triathlon could be increased systemic inflammation after Ironman distance triathlons [19,20,21,22] compared to after marathons [29]. A case study of one subject after completing a 25-day ultra-endurance exercise challenge shows a slight reduction in FMD as long as 48 h after completion [16], and ultra-endurance exercise is also known to cause significantly increased levels of biomarkers for inflammation [30]. In the present study, we found increased levels of the endothelial adhesion molecules E-selectin, ICAM-1, and VCAM-1 after the Ironman distance triathlon, along with reduced FMD. These endothelial adhesion molecules are upregulated due to inflammation [25], but can also be modulated by increased shear stress [31]. There is evidence from cardiovascular disease patients of an inverse correlation between E-selectin levels and FMD-response [32] supporting a possible direct effect between the adhesion molecules and the reduced FMD. On the other hand, increased levels of E-selectin, ICAM-1, and VCAM-1 were demonstrated after the Oslo Marathon [33] and in another study, the researchers found no decrease in brachial FMD after the London Marathon [6], suggesting a lack of direct effect between the endothelial adhesion molecules and FMD. However, both of these studies were conducted after marathons with primary usage of only the lower body as discussed above. We measured circulating levels of soluble endothelial adhesion molecules, and we do not know in which part of the arterial tree these molecules are expressed. We assume that there is a larger accumulated dose of shear stress in the brachial artery after a triathlon than after a marathon due to the nature of the upper body’s usage in triathlons [28]. Therefore, it is possible that increased expression of the adhesion molecules locally in the brachial artery can partly explain the reduced FMD observed in our study. Endothelial inflammation causes reduced NO-secretion through cytokine-stimulated intracellular production of reactive oxygen species (ROS) that reacts with NO, known as NOS-uncoupling [34]. FMD is partly NO-dependent [4] and reduced levels of NO could therefore explain the reduced FMD response in a state with endothelial inflammation. A recent article showed significantly increased ROS production rates in capillary blood after completing an ironman distance triathlon [35], supporting this hypothesis.

Reduced circulating L-arginine levels could offer a mechanistic explanation for the reduced FMD-response upon completion of the race. Vascular endothelial cells synthesize NO from L-arginine by NO synthase (NOS) [24,31], predominantly through endothelial NOS (eNOS) in endothelial cells [36]. The K_m_ (concentration at which the reaction rate is half of its maximal value) is 2.9 μM for purified eNOS [37], which is far less than the observed 35.8 (34.3–38.1) μM. However, experiments on living bovine endothelial cells show that L-arginine uptake into the intracellular compartment containing eNOS is a limiting factor and demonstrate a K_m_ of 29 ± 6 μM calculated by extracellular L-arginine levels effect on intracellular eNOS activity [38]. Assuming similar circulating and extracellular levels of L-arginine, this puts measured circulating levels after the race within the range one can expect to be significant for intracellular eNOS activity. There are several studies of the effect of increased circulating L-arginine through food supplements on FMD with varying results ranging from no effect on FMD [39,40] to improved FMD [41,42,43] and varying effects dependent on baseline values in overweight adults [44]. All of these studies [39,40,41,42,43,44] examine the effects of increased L-arginine from values around 50–120 μM at baseline in different populations with further increased values after using supplements. Therefore, one would expect less significance for NO production at these concentrations based on the in vitro studies of the K_m_ for eNOS [38]. There are no studies to our awareness that examine FMD with acutely reduced L-arginine, as seen in our study. Therefore, we believe it is possible that reduced levels of L-arginine below levels of 40 μM, as seen in our study, could partly explain impaired endothelial function as measured with reduced FMD.

In addition to increased endothelial inflammation and reduced L-arginine levels, other possible mechanisms not evaluated in the present study might reduce FMD post-exercise. These include increased sympathetic activity [45,46] and variations due to measurements at different times of the day [47]. Both baseline diameter [48] and altered flow stimulus [49] can alter the FMD response. However, we did not find any significant differences in baseline diameter or flow between the measurement times.

One week after the race, we found VCAM-1 to be slightly reduced compared to baseline, while there was no significant change between baseline and the measurement one week after for FMD, ICAM-1, E-selectin, and L-arginine. We do not have a biochemical answer for the reduced VCAM-1 levels one week after the race. There is no international reference for VCAM-1, but the producer of the ELISA kits reports a standard range of 350–990 ng/mL for VCAM-1, and the levels one week after the race were within these limits. A limitation in the present study is the relatively small sample size, making it possible that there were other minor changes after the race that we did not find statistically significant due to a lack of statistical power. However, the more considerable changes found immediately after the race was not present one week later.

The significantly reduced endothelial function with increased endothelial inflammation and reduced L-arginine bioavailability was transient. Therefore, there was no evidence of any long-lasting reduced endothelial function after an Ironman triathlon from our study. However, it was previously proposed that there may be a U-shaped effect of exercise on endothelial function over time. It was proposed that moderate to high levels of exercise have a beneficial effect on endothelial function while both lack of exercise and extreme levels of exercise cause impaired endothelial function in otherwise healthy individuals [50]. Supporting this thesis there is evidence of an increased prevalence of subclinical coronary artery calcification and plaques in male long-distance runners compared to matched controls [51,52]. Therefore, we suggest that repeated episodes of transient endothelial inflammation and reduced endothelial function, such as demonstrated in this study, might be a part of the pathogenesis for the development of arterial calcification in long-distance endurance athletes.

## 5. Conclusions

The reduced FMD and L-arginine levels found immediately after completing the Norseman Xtreme triathlon were normalized one week after. In parallel, there was an altered endothelial inflammation that might in part explain the altered endothelial function.

## Figures and Tables

**Table 1:** Characteristics of the subjects. Values are given as median (1. quartile, 3. quartile). N = 9; * Reported average weekly exercise last year. ** Reported number of years competing in long-distance running or triathlons, marathon distance or above. All blood pressures were measured at rest before the race.

Characteristic	Value
Age (years)	43 (40–49)
Weight (Kg)	78 (70.6–78.5)
Height (m)	1.81 (1.74–1.83)
Weekly endurance exercise * (h)	16 (12–18)
Weekly strength exercise * (h)	1 (0–2)
Completed Ironman triathlons (n)	1 (0–4)
Completed marathons (n)	5 (1–7)
Completed ultra-distance races (n)	2 (0–7)
Years of competing ** (y)	7 (5–10)
Body mass index (Kg/m^2^)	23.4 (22.1–24.7)
Systolic blood pressure (mmHg)	127 (125–130)
Diastolic blood pressure (mmHg)	73 (70–78)
Swim time (h)	1.1 (1.0–1.3)
Bike time (h)	7.0 (6.4–7.3)
Run time (h)	5.9 (5.3–6.2)
Finish time (h)	14.5 (13.4–15.3)

## Data Availability

The data presented in this study are openly available in the Supplemental Information as S1. Race times and the age of the subjects are omitted in the raw data to ensure anonymity for the subjects, as the race results are public.

## Supplementary Materials

The following are available online at https://www.mdpi.com/article/10.3390/sports9060086/s1, S1: Raw data. CSV-file containing the raw data from the research.
